# Understanding Variation in Transcription Factor Binding by Modeling Transcription Factor Genome-Epigenome Interactions

**DOI:** 10.1371/journal.pcbi.1003367

**Published:** 2013-12-05

**Authors:** Chieh-Chun Chen, Shu Xiao, Dan Xie, Xiaoyi Cao, Chun-Xiao Song, Ting Wang, Chuan He, Sheng Zhong

**Affiliations:** 1 Department of Bioengineering, University of Illinois at Urbana-Champaign, Urbana, Illinois, United States of America; 2 Department of Bioengineering, University of California San Diego, La Jolla, California, United States of America; 3 Department of Chemistry, University of Chicago, Chicago, Illinois, United States of America; 4 Department of Genetics, Washington University in St. Louis, St. Louis, Missouri, United States of America; Max-Planck-Institut für Informatik, Germany

## Abstract

Despite explosive growth in genomic datasets, the methods for studying epigenomic mechanisms of gene regulation remain primitive. Here we present a model-based approach to systematically analyze the epigenomic functions in modulating transcription factor-DNA binding. Based on the first principles of statistical mechanics, this model considers the interactions between epigenomic modifications and a cis-regulatory module, which contains multiple binding sites arranged in any configurations. We compiled a comprehensive epigenomic dataset in mouse embryonic stem (mES) cells, including DNA methylation (MeDIP-seq and MRE-seq), DNA hydroxymethylation (5-hmC-seq), and histone modifications (ChIP-seq). We discovered correlations of transcription factors (TFs) for specific combinations of epigenomic modifications, which we term epigenomic motifs. Epigenomic motifs explained why some TFs appeared to have different DNA binding motifs derived from *in vivo* (ChIP-seq) and *in vitro* experiments. Theoretical analyses suggested that the epigenome can modulate transcriptional noise and boost the cooperativity of weak TF binding sites. ChIP-seq data suggested that epigenomic boost of binding affinities in weak TF binding sites can function in mES cells. We showed in theory that the epigenome should suppress the TF binding differences on SNP-containing binding sites in two people. Using personal data, we identified strong associations between H3K4me2/H3K9ac and the degree of personal differences in NFκB binding in SNP-containing binding sites, which may explain why some SNPs introduce much smaller personal variations on TF binding than other SNPs. In summary, this model presents a powerful approach to analyze the functions of epigenomic modifications. This model was implemented into an open source program APEG (Affinity Prediction by Epigenome and Genome, http://systemsbio.ucsd.edu/apeg).

## Author Summary

We developed a model-based approach to systematically analyze the epigenomic functions in modulating transcription factor-DNA binding. We postulated the existence of TF-specific epigenomic motifs, which could explain why some TFs appeared to have different DNA binding motifs derived from *in vivo* and *in vitro* experiments. The theoretical results suggested that the epigenome can modulate transcriptional noise and boost the cooperativity of weak TF binding sites. A preliminary analysis of the existing data suggested that epigenomic boost of binding affinities in weak TF binding sites could be a widespread regulatory mechanism in mES cells. Moreover, using personal data, we identified strong associations between H3K4me2/H3K9ac and the degree of individual differences in NFκB binding in SNP-containing binding sites, suggesting the theoretical mechanism for epigenome to attenuate the TF binding differences on SNP-containing binding sites in two individuals may contribute to link genomic variation to phenotypic variation. Thus, this model presents a powerful approach to analyze the functions of epigenomic modifications.

## Introduction

Central to transcriptional regulation of gene expression is the regulation of the quantities of transcription factors (TF) bound to genomic regulatory sequences. The information used to quantitatively control TF-DNA binding is not only encoded in the genomic sequences, but likely is also embedded in the chemical modifications to the genomic sequences and the nearby histones [1]. The chemical modifications (called epigenomic modifications) include the addition of a methyl group or a hydroxymethyl group to the 5th carbon of cytosine (5-mC and 5-hmC) and a number of posttranslational modifications to the histone proteins [2]. These modifications can alter the chromatin structure and function by changing the charge of the nucleosome or directly interacting with TFs [3]. In turn, TFs can tether DNA modification enzymes and histone modification enzymes to change the epigenomic modifications around the TF binding region. Hence, both the genomic sequences and the epigenetic modifications contribute to define the regional diversity of the regulatory genome. Less clear is how the genome and the epigenome jointly encode regulatory information, and how TFs interact with such regulatory information. The goal of this work is to model the three-way interactions among the TFs, the genomic sequence, and the epigenome, and thus allowing for predicting TF binding affinities in equilibrium states.

Genome-wide distributions of TF-binding and epigenomic modifications can now be obtained by high-throughput sequencing methods [4]. The explosive growth of data urges the methodological developments that can achieve mechanistic understanding of gene regulation. In particular, quantitative models are needed to learn the regulatory rules implemented by epigenomic modifications. Two classes of methods were developed to study transcriptional regulation with different goals and mathematical foundations. The first class of methods aims at deriving regulator-target relationships or finding regulatory sequences and motifs. These methods were built upon statistical associations among sequence patterns, TF binding, and gene expression [5]–[11]. An advantage of this class of methods is that it is easy to incorporate new data types including epigenomic modifications. Indeed, using statistical enrichment and machine learning ideas, recent efforts have incorporated nucleosome positions [12] and epigenomic modifications to identify TFBSs [13] and regulatory genomic sequences [12], [14]–[18] (Table S1B). However, machine learning methods do not allow direct biophysical interpretation for their parameters, and therefore they do not make biological inferences as directly as the thermodynamic models (see below).

The second class of methods aims at deriving molecular mechanisms of TF-DNA interactions, using a thermodynamic framework (reviewed in [19]). The intensity of TF-DNA binding was modeled as the equilibrium output of input sequences and TFs [20], [21]. Partially due to a huge computational burden, this class of methods was originally restricted to analyze a few selected regulatory sequences in single-cell organisms, where a few simplified assumptions can be made [20]–[22] (Model assumptions, Table S1A). These models were extended to analyze nucleosome positions [23], [24], gene expression in *drosophila* embryonic development [25]–[27], and genome-wide TF binding data [28]. The latter development offered a unique advantage, which is the capability of gaining mechanistic understanding of TF-TF interaction and TF-DNA binding from genome-wide binding data. However, this class of models cannot easily take into account epigenomic modifications, which are argued to be more influential to TF-DNA binding than cooperative interactions between TFs [29], [30]. Here we present a thermodynamic model that incorporates epigenomic modifications. This model can learn synergistic and antagonistic interactions between specific TFs and epigenomic modifications from genome-wide TF binding and epigenomic data.

We were interested in a few open questions on the mechanisms of TF-DNA binding. First, to what extent does an epigenetic modification change the binding strength between a TF and a genomic sequence, which is composed of multiple strong and weak binding sites? Second, is the epigenomic influence to TF-DNA binding invariant to the nucleotide composition of the genomic sequence? Third, many TFs have preferred DNA recognition codes (a.k.a. motifs); are there TF-specific epigenomic recognition codes? Fourth, does the epigenome modulate the variability (noise) of gene expression in an isogenic cell population? Finally, what is the role of the epigenome in modulating individual variation of TF-binding among humans?

We used two complementary experimental systems to study the above questions. The first system is mouse embryonic stem (mES) cells. We recently assayed genome-wide distributions of 5-methylcytosine (5-mC), 5-hydroxymethylcytosine (5-hmC), histone variant H2A.Z, and acetylation of histone 3 lysine 27 (H3K27ac) [31]. We combined these data with published chromatin immunoprecipitation followed by sequencing (ChIP-seq) datasets of 5 other epigenomic modifications [2], [32], [33] and 9 TFs [34] from mES cells. This combined dataset allowed us to study TF-epigenome-DNA interactions relatively comprehensively. The second system is the white blood cells of seven people, which allowed us to explore individual differences in humans.

## Results

### An epigenome-sensitive TF-DNA binding model

**Figure 1:**
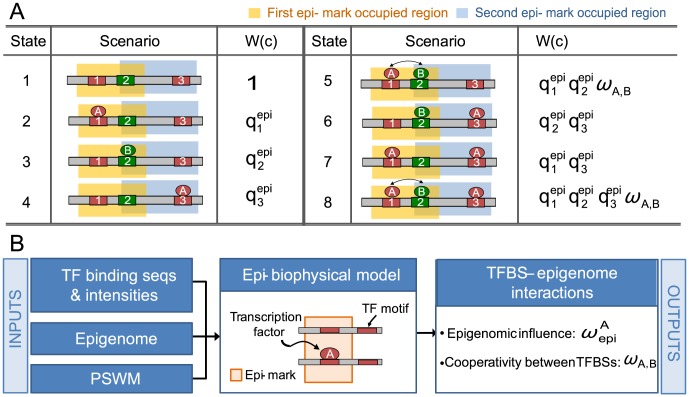
Modeling the epigenome and the genome as a physical system. (A) The states of the system and their probabilities. As an example, a hypothetical genomic sequence is occupied by two epigenomic modifications (orange and gray shades), which partially overlap. The sequence contains three TFBSs for two TFs (A and B). The two TFBSs for A (red boxes) are each occupied by one epigenomic modification, and the TFBS for B (green box) is located in the overlapping region of the two modifications. The first TFBS for A (red box on the left) and the TFBS for B are close enough for their bound TFs to interact (arrows in States 5 and 8). Because each of the three TFBSs can reside in either the bound or the unbound state, the whole sequence can reside in a total of 2^3^ physical states (listed in the State column). *c*: a physical state; *W*(*c*): Boltzmann weight for state *c*, which is proportional to the probability that the system visits this state; *q^epi^*: the binding affinity between a transcription factor and the sequence under the epigenomic context. (B) The workflow for inferring epigenetic marks that influence the binding of a TF. Central to this workflow is our epigenome-sensitive TF-DNA binding model (the Epigenetic biophysical model). Inputs to this model are TF binding data (ChIP-seq), PSWM of the TF and epigenomic modification data (ChIP-seq, 5-hmC-seq, MeDIP-seq, and MRE-seq). Outputs of the model include the influences of epigenomic marks to the binding of each transcription factor and the cooperativities between TFBSs.

We developed a quantitative model for TF-DNA binding in a given epigenomic context. The goal of this model is to predict the binding intensity of a TF in any genomic region in any cell type, using the genomic sequence and the epigenomic modifications (cell-type-specific data). This model incorporates four types of biophysical information: the active concentrations of the TFs (learned from ChIP-seq data), the binding preferences of these TFs to DNA (motif), the nucleotide composition of the genomic sequence, and the epigenomic modifications (see Methods). Given input data including position-specific weight matrices (PSWM), ChIP-seq derived TF binding sequences and binding intensities, and genome-wide distribution of epigenomic modifications, this model can learn cooperativity among TFBSs (any number of strong and weak, homotypic and heterotypic TFBSs). More importantly, it can learn synergistic and antagonistic interactions between a specific TF and every assayed epigenomic modification. The learning process involves two steps (Figure 1B). First, the model scans each epigenomic mark independently to identify those that interact with the transcription factor of the interest and modulate its binding affinities to genomic sequences. Second, these identified epigenomic marks are combined into one unified model to predict the binding affinity of any genomic regions. The model quantifies the improvements of predicted binding affinities by using the identified epigenomic marks (Table S2). Because this model operates at thermodynamic equilibrium, it does not make causal inferences about epigenetic and TF binding changes. We implemented this model into an open source program APEG (Affinity Prediction by Epigenome and Genome, http://systemsbio.ucsd.edu/apeg).

### Genome-wide distributions of 5-methylcytosine, 5-hydroxymethylcytosine, H2A.Z, and H3K27ac in mES cells

We recently published two types of 5-methylcytosine (5-mC) data in E14 mES cells, using methylated DNA immunoprecipitation followed by sequencing (MeDIP-seq) and DNA digestion by methyl-sensitive restriction enzymes followed by sequencing (MRE-seq) [31], . A total of 45.2 million reads were generated from MeDIP-seq, reflecting 1,495,114 methylated 200 bp genomic segments (windows) across the genome (Text S1). A total of 2.1 million MRE-seq reads were generated from a total of three restriction enzymes, covering 428,367 unmethylated windows. We used a selective chemical labeling method to pull down and sequence 5-hydroxymethylcytosine (5-hmC) regions (5-hmC-seq) [31], [36]. A total of 58 million 5-hmC-seq sequence reads were generated, detecting 1.5 million 5-hmC marked windows in mES cells. We assayed the genomic distribution of histone variant H2A.Z and acetylation of Histone 3 Lysine 27 (H3K27ac) in E14 mES cells [31]. With 19.9 million ChIP-seq reads, 1.1 million 200 bp windows were found to contain H2A.Z. It has a small overlap with promoter regions (10.45% of H2A.Z marked windows), suggesting its substantial involvement in distal regulatory regions [37]–[39]. With a total of 19.8 million ChIP-seq reads, around 1.0 million 200 bp windows were marked by H3K27ac. It had a moderate overlap with promoter regions (16.66%), in line with the thought that it is primarily an enhancer mark [40]. Interestingly, H2A.Z and H3K27ac exhibited differential overlaps with 5-hmC marked windows (25.69% and 31.57%) and 5-mC marked windows (17.69% and 12.98%), respectively. This suggests both H2A.Z and H3K27ac tend to overlap with 5-hmC more than with 5-mC (both p-values<2.2e-16, Chi-square test). Combining these data with 6 published ChIP-seq datasets [2], [32], [33], we obtained genome-wide distributions of 9 epigenetic marks and 9 transcription factors in mES cells, which served as the dataset for our model-based analyses.

### Identification of TF-specific epigenomic motifs

**Figure 2:**
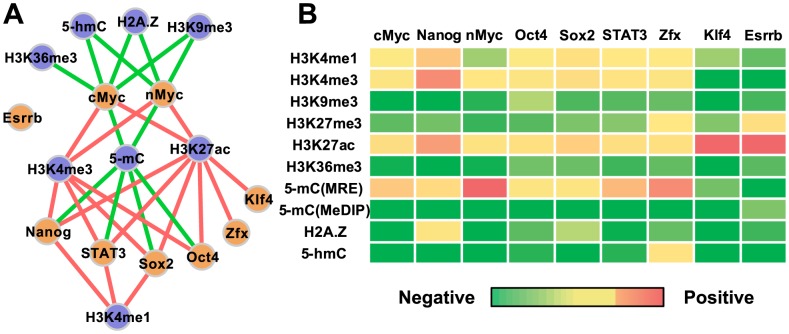
Transcription factor-specific epigenomic codes. (A) An interaction network between TFs (orange nodes) and epigenetic modifications (blue nodes) in mES cells (p-value cutoff = 0.05). The interactions include positive (red edges) and negative correlations (green edges) of TF binding and epigenetic marks. This network suggests that each TF has its specific epigenetic marks for interaction. (B) TF-specific epigenomic motifs. The influences of every epigenetic mark to the binding of a TF (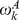 in Equation (5)) are summarized as a column vector. In analogy to matrix presentation of DNA recognition motifs, we propose to use a column vector to represent the epigenomic motif of a TF. Each column represents an epigenomic motif {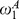, …, 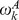}, and the first column is {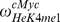, …, 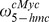}.

Even though some epigenomic modifications are assumed to take some general roles in synergizing or antagonizing TF-DNA binding, little is known whether such epigenomic functions are specific to certain TFs or are general to every TF. To explore this question, we applied our new model to genome-wide distribution data of 9 TFs and 9 types of epigenomic modifications in mES cells (assayed by ChIP-seq, MeDIP-seq, MRE-seq, and 5-hmC-seq). Thirty interactions between TFs and epigenomic modifications were identified, forming an interaction network (Figure 2A, Table S2). Here, “interaction” refers to the positive or negative correlation of an epigenetic modification and the binding between a TF and DNA. Among the 9 epigenetic modifications, H3K4me3, H3K27ac, and 5-mC each interacts with a large number of TFs, forming hubs in the interaction network. Among the five epigenetic modifications that exhibited negative roles, only 5-mC represses the mES cell-specific regulators Oct4, Sox2, Nanog, and Stat3. Compared to the hubs, H3K4me1 is more specific. It plays a positive role to the binding of Nanog, Sox2 and Stat3. Even more specific are H2A.Z, 5-hmC, and H3K9me3, which may negatively correlate with the binding of cMyc and nMyc. These data suggest that not all epigenomic modifications “uniformly” interact with every TF. Some epigenetic modifications may be associated with the binding of specific TFs.

Considering TFs often have recognition preferences to certain short genomic sequences (motifs), we hypothesized that there are TF-specific epigenomic motifs. By an epigenomic motif we refer to a specific combination of epigenetic modifications that is characteristic to the in vivo binding sites of a TF. To test this hypothesis, we estimated the association of every epigenetic modification and the binding of each TF, i.e. 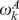 in Equation (5). For each TF, we compiled the influences of epigenetic modifications as a column vector (Figure 2B). These influences are not identical across TFs (columns of Figure 2B). This suggests that analogous to DNA motifs, in vivo TF-DNA binding also have epigenomic motifs. A PSWM is used to describe DNA motifs [41]. We propose to use the vector of model-learned influences of the K epigenetic marks {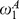, …, 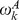} to describe TF-specific epigenomic motifs, where A denotes the TF of our interest and K represents the total number of epigenetic marks. The epigenomic motifs can be used in combination with PSWMs to approximate the binding preferences of transcription factors *in vivo*.

### Epigenomic motif improves predictions of TF binding intensities

**Figure 3:**
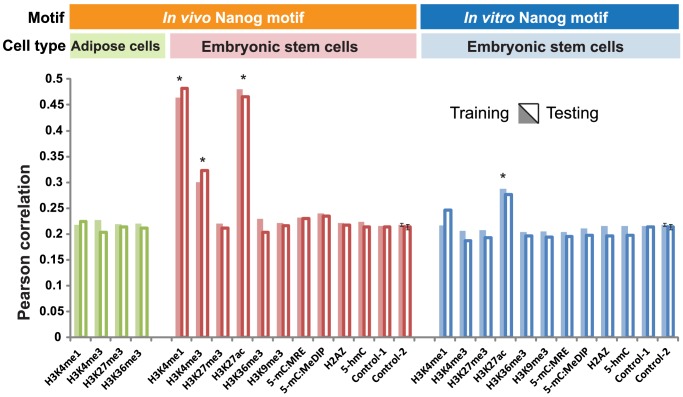
Epigenomic marks improve model predictions of Nanog binding. Model predicted binding intensities are correlated to ChIP-seq reported binding intensities (y axis: Pearson correlation). The model predictions are based on sequence data alone (Control-1), sequence data plus randomized epigenomic data (Control-2), or sequence data plus one epigenomic mark (other columns). Results on both training data (shaded bars) and testing data (hollow bars) are plotted. Epigenomic marks that significantly improve the predictions of Nanog binding (marked by *) are identified by using the standard deviations of the control experiments (error bars). Combined with the Nanog motif (PSWM) derived from *in vivo* experiments (red bars), several epigenomic marks can increase the accuracy of predicted binding intensities, achieving Pearson Correlations above 0.47 (H3K4me3 and H3K27ac, red bars). However, combined with the Nanog motif derived from *in vitro* experiments (blue bars), no epigenetic mark except H3K27ac can improve the predictions of ChIP-seq measurements. Even for H3K27ac, the Pearson Correlation obtained from the *in vitro* motif (0.29) is much smaller than the Pearson correlation obtained from the *in vivo* motif (0.47). None of the four measured epigenomic marks in adipose cells help to better predict Nanog binding in stem cells (green bars), suggesting that cell-type-specific epigenetic data are required for increasing the prediction accuracy.

We hypothesized that the predictive power of TF binding intensities should be increased by incorporating the information of epigenomic motifs. In other words, if epigenomic motifs exist, they should help to better predict TF binding intensities than using DNA sequences alone. Three computational experiments were done to test this hypothesis. We chose the Nanog TF for these experiments, mostly because Nanog is an essential TF in ES cells and Nanog's DNA recognition motif is not well understood. In the first experiment, we removed the epigenomic data and fed our model with genomic sequences only. Without epigenomic data, our model degenerates into the STAP model [28]. STAP uses the sequences (500 bp) and the TF-specific PSWM to predict TF binding affinities, taking into account all possible interactions among strong and weak TFBSs. To quantify the model's predictive power, we used the Pearson correlation between the ChIP-seq signals (as observed binding intensities) and the model-predicted binding intensities. Pearson correlations were 0.211 and 0.212 in the training and the testing datasets, respectively, providing a baseline predictive power (Control-1 in red, Figure 3). We then applied the model to test each epigenomic modification. H3K4me1, H3K27ac and H3K4me3 largely increased the model's predictive power of Nanog binding intensities from the baseline (red bars, Figure 3). These three epigenomic marks were thus inferred as interacting with Nanog. To test the robustness of model inference, we changed the metric for quantifying prediction power into Spearman's rank correlation (Figure S1) and varied window sizes (Figure S2). Neither of these changes affected the inferred interacting epigenomic marks.

In the second experiment, we randomly shuffled the genomic positions of the observed epigenetic modification intensities, generating 200 permutated datasets. Feeding the permutated datasets to the model, we obtained a background distribution of predictive power (Control-2 in red, Figure 3). Using this background distribution, we identified three epigenetic modifications with which the model can significantly better predict TF binding intensities (red bars with * in Figure 3, permutation p-value = 0). These three epigenetic modifications were identified as interacting with Nanog. This permutation experiment used the same number of model parameters and the same amount of data (PSWM, sequence, and epigenetic data) as the experiment using the original data. It rules out the possibility that the increased predictive power was due to increased model complexities.

### TF-specific epigenomic motif is cell-type specific

As the 3^rd^ control experiment, we replaced the epigenetic modifications in mES cells with the epigenetic modifications of mouse adipose cells [42] and kept the other data intact. None of the four epigenetic modifications in mouse adipose cells significantly increased the predictive power of Nanog binding in mES cells (green bars vs. Control-1 and Control-2 in red, Figure 3), suggesting our learned TFBS-epigenomic interactions were cell-type specific.

### Epigenome alone is less predictive of TF binding than epigenome and genome combined

We asked to what extent the epigenome can predict TF binding without using the genomic sequences. Two control datasets were generated. First, each epigenomic mark was fed to our model without sequence data (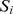 becomes invariant to 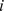 in Equation (4), solid red bars, Figure S3). The enhancer and open chromatin marks H3K4me1 and H3K27ac were most strongly predictive of Nanog binding, followed by the promoter mark H3K4me3. These data are consistent with the idea that open chromatin and hypersensitivity sites are predictive of transcription factor binding regions [18]. Interestingly, H2A.Z is the fourth epigenomic mark that is predictive of Nanog binding. The regulatory function of H2A.Z in mammalian cells remains controversial. While H2A.Z is generally thought as an active mark of transcription, it is negatively correlated with gene expression in a mES cell differentiation process [43]. The positive association of H2A.Z with Nanog binding suggests that H2A.Z may facilitate Nanog binding in undifferentiated mES cells. Second, we collected all (214) PSWMs from the JASPAR database as background motifs [44]. These background PSWMs were fed to the model with each epigenomic mark. The mean and standard deviation of the model predicted binding intensities from these background PSWMs were derived (hollow red bars and error bars, Figure S3). The predictive powers of these control datasets were compared to the predictive powers using both epigenomic and PSWM information (blue bars, Figure S3). The *in vivo* Nanog motif combined with epigenomic data (solid blue bars) increased the accuracy of predicted Nanog binding affinities than using epigenomic data alone (red bars). More than 20% increases of predictive power were observed using Nanog motif and H3K4me1 or H3K27ac than using H3K4me1 or H3K27ac alone. Even larger increases were found in comparing *in vivo* Nanog motif (solid blue bars) with background PSWMs (hollow red bars). The latter comparison used models with the same number of model parameters. It rules out the possibility that the increased predictive power was due to increased model complexities.

### *In vitro* derived TF-DNA binding motifs do not interact with epigenomic motif

The TF-DNA binding motifs derived from the enriched sequence patterns using *in vitro* binding assays do not always agree with the enriched motifs from *in vivo* binding assays [45]. Depending on the TFs, the differences in motifs derived from *in vitro* and in *vivo* experiments can be small [46] or large [28]. The causes of such differences are unknown. We hypothesized that some epigenomic modifications can synergize with DNA to produce a somewhat different binding preference of a TF than the binding preference of this TF to naked DNA. To test this hypothesis, we chose to further analyze the Nanog motifs derived *in vitro* [47] and *in vivo* [28]. We used the *in vitro* Nanog motif together with all epigenomic data to learn and predict *in vivo* binding affinities (blue bars, Figure 3) and compared to the results from the *in vivo* motif (red bars, Figure 3). Without considering epigenomic data, the *in vitro* and *in vivo* motifs had similar predictive powers of ChIP-seq signals (Control-1 in red vs. Control-1 in blue, Figure 3). However, except for H3K27ac, adding epigenetic modifications to the *in vitro* motif did not increase the predictive power of Nanog binding. Even for H3K27ac, its contribution to predicting Nanog binding was much larger when combined with the *in vivo* motif than when combined with the *in vitro* motif (red and blue H3K27ac bars, Figure 3). This means the model failed to identify clear TFBS-epigenomic interactions with the *in vitro* Nanog motif, suggesting that the epigenomic motif is specific to the *in vivo* Nanog DNA binding motif. In several cases, including H3K4me3, H3K27me3, H3K36me3, and 5-mC (both MRE and MeDIP), feeding the model with epigenetic data together with the *in vitro* motif even slightly decreased its predictive power as compared to not using epigenetic data at all (blue bars vs. Control-1 in blue, Figure 3). This is because the model allowing for TFBS-epigenomic interactions is more complex than that without epigenetic data. However, there is no extra information added due to the lack of interaction between the *in vitro* motif and the epigenetic marks. These data explain the difference between the TF-DNA binding motifs derived *in vivo* and *in vitro*: although the Nanog sequence motifs derived *in vitro* and *in vivo* have similar binding affinities to the Nanog protein *in vitro* [28], the *in vivo* motif predicted Nanog binding events with a higher sensitivity given the specificity (Figure S4). This suggests that only the *in vivo* motif may interact with epigenetic modifications. The *in vivo* binding intensities are determined by TFBS-epigenomic interactions and cannot be faithfully reproduced with the sequence motif (either *in vitro* or *in vivo*) alone.

### Epigenomic regulation of transcriptional noise

We asked how epigenomic modifications may theoretically modulate transcriptional noise [48] and the cooperativity of TFBSs. To address this question, we used constraint-based simulation studies [49], with the constraints being the physical and empirical limits of TF concentrations and epigenomic states in eukaryotic cells (Text S1).

Transcriptional noise is the variability of gene expression among cells in an isogenic population [48], [50], [51]. We asked whether the epigenome can modulate the level of transcriptional noise. We studied simple transcription systems with one TFBS, by examining the change in binding probability as a function of the concentration of the TF and the presence of epigenomic marks. Following the main assumption of thermodynamic models of gene expression, every cell in an isogenic cell population has the same probability of producing a transcript, denoted as *p* (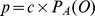, where 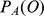 is defined in Equation (1) and 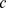 is a constant). The expected number of transcripts is proportional to 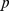, therefore the variability of 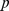 reflects transcriptional noise [23].

**Figure 4:**
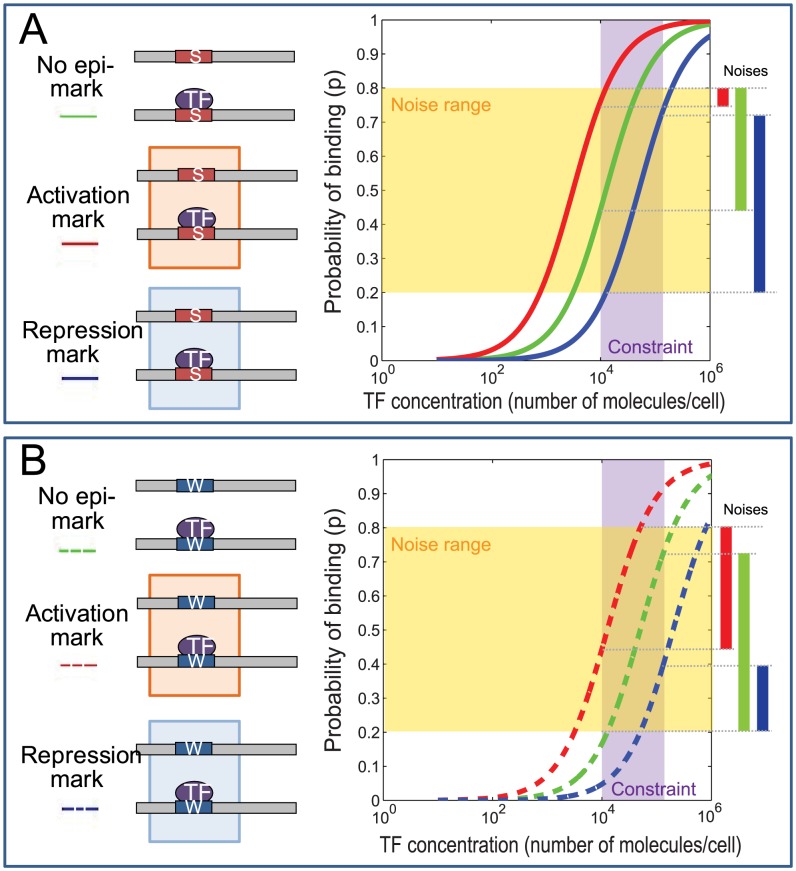
Epigenomic regulation of transcriptional noise. Transcriptional noise is introduced when the binding probability (y axis) between a TF and its target TFBS falls into a particular range (horizontal yellow band). There is nearly no noise above or below this range, because almost all cells would uniformly have this target TFBS in the bound or the unbound state, respectively. The binding probabilities are constrained by the realistic range (vertical blue band) of TF concentrations in eukaryotic cells (x axis). (A) In the presence of a strong binding site (S), the binding probabilities are shown as functions of the TF concentration and the presence of epigenomic marks (Red curve: activation mark, green: no epigenomic marks, blue: repression mark). Activation marks suppress transcriptional noise by reducing the range of feasible binding probabilities, whereas repression marks enhance transcriptional noise. (B) In the presence of a weak binding site (W), both activation (red) and repression (blue) marks tend to suppress transcriptional noise.

Without any epigenomic marks, the binding probability increased as the concentration of the TF increased, forming a sigmoid curve (green curve, Figure 4A–B). In a transcriptional system with one strong TFBS, the binding probability should reach the half of the maximum binding probability when the TF concentration passes a low threshold [52]. With a weak TFBS, the half of maximum binding probability should be reached at a high threshold of the TF concentration. Because the range of TF concentrations is generally between 10,000 and 300,000 molecules per cell in fruit fly, mouse, and human cells (reviewed by [29]), in our simulation of a strong TFBS, the half of the maximum binding probability was reached when the TF concentration reached 10,000 molecules per cell (green curve, Figure 4A). In the other simulated system containing a weak TFBS, the half of the maximum binding was reached at the TF concentration of 300,000 molecules per cell (green curve, Figure 4B).

In the presence of an activation mark, the sigmoid curve of binding probabilities shifted to the left (red curve, Figure 4A–B) with no overlap to the original curve. Similarly, in the presence of a repression mark, the curve shifted to the right (blue curve, Figure 4A–B). The dynamic range of TF binding probabilities, constrained by the range of TF concentrations, is a major indicator of transcriptional noise [23]. These constraint-based simulations provided a theoretical prediction that in the presence of a strong binding site, an activation mark decreases the dynamic range of binding probabilities and thus suppresses transcriptional noise, whereas a repression mark enhances transcriptional noise (Figure 4A). However, in a transcriptional system with a single weak binding site, both activation and repression marks tend to suppress transcriptional noise (Figure 4B). The key assumption to these predictions is that the half of total binding probability of a weak (strong) TFBS is reached at about the upper (lower) bound of the available concentrations of the TF.

### The epigenome may boost the cooperativity of weak binding sites

**Figure 5:**
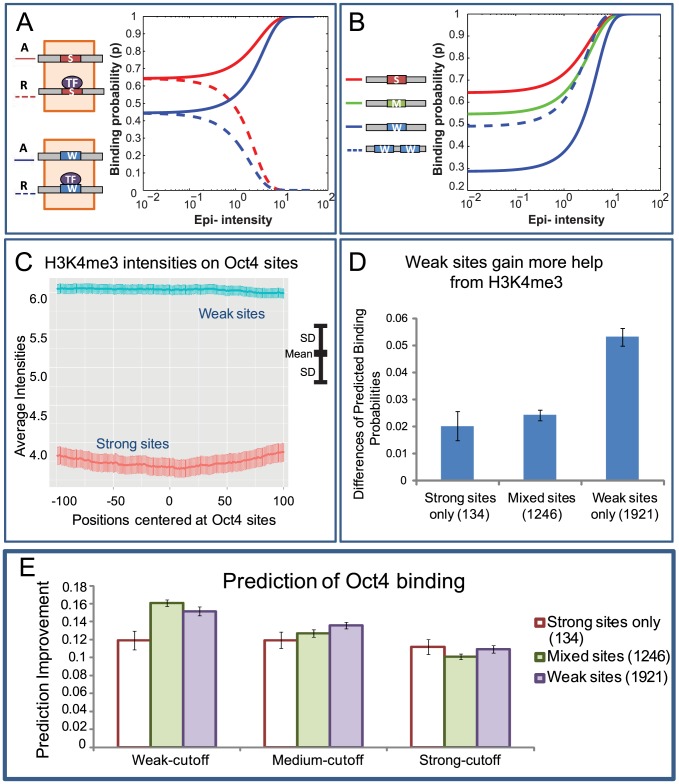
Epigenomic boost of cooperativity of weak binding sites. (A) The relationship between binding probability and epigenetic intensity. Given the transcription factor concentration, the binding probability (y axis) is shown as functions of the intensities (Epigenetic intensity, x axis) and types (solid: activation, dashed: repression) of epigenomic modifications and the strengths of the binding sites (red: strong, blue: weak). For a single binding site, the binding probability is monotonic to the strength of the binding site for all intensities of epigenomic modifications (red curves are always above blue curves). (B) Epigenomic boost of binding-site cooperativity. In the presence of an activation mark, the binding probabilities are monotonic for single strong (red), medium-strength (green), and weak (blue) binding sites. A pair of two weak binding sites has a smaller binding probability in the absence of the activation mark (dotted blue line at Epigenetic intensity = 10^−2^). While the intensity of the activation mark increases, the binding probability of this pair of weak sites gradually surpasses that of the medium-strength site and the strong binding site, breaking the monotonicity of single binding sites. (C) Activation mark H3K4me3 has larger average intensities in weak-TFBS regions (blue) than in strong-TFBS-containing regions (red). SD: standard deviation. (D) The difference of model-predicted binding probabilities with and without the epigenome (y axis) is larger in weak-TFBS-only regions (right column) than in the regions containing both strong and weak sites (mixed, middle column), which in turn is larger than in the strong-TFBS-only regions (left column). (E) H3K4me3 enables larger improvements of prediction accuracy on regions containing weak TFBSs. The predictions of binding for these regions were determined by applying three cutoffs on the predicted binding probability calculated from the model (Weak-cutoff (0.3), Medium-cutoff (0.35), Strong-cutoff (0.4)). The improvement of prediction accuracy was quantified by comparing the predictions by sequence and H3K4me3 to the predictions by sequence only (Y-axis). The improvement was defined as the difference of the proportions of correctly predicted binding regions between using and not using H3K4me3 data. Red, purple, and green bars represent the sequences that contain strong, weak, and mixed TFBSs as determined by PSWM matching scores. Error bars: standard deviations. The number of each type of sequences is in parenthesis.

We asked whether the epigenome could modulate the cooperativity of adjacent TFBSs. To obtain a baseline (no cooperativity) for this analysis, in a simulation study, we fixed the TF concentration ( [A] in Equation (4)) and compared the binding affinities between a strong TFBS and a weak TFBS in various epigenomic conditions. As expected, in the presence of an activation mark, the binding affinity increases as the intensity of this activation modification increases (solid curves, Figure 5A), and the reverse is true in the presence of a repression mark (dashed curves, Figure 5A). Moreover, an increase of epigenomic intensity produces a smaller difference in the binding affinities of the two TFBSs (solid and dashed curves become closer as epigenomic intensity increases, Figure 5A). However, the binding affinity of a weak TFBS cannot surpass the affinity of a strong TFBS in any levels of an epigenomic modification (neither the solid curves nor the dashed curves crossed, Figure 5A). In other words, when there is no cooperativity between TFBSs, under the same epigenomic condition, the order of binding strengths among different genomic sequences is fixed. Because TF concentration ([A]) is a multiplicative factor that is separate from the rest in the calculation of the binding affinity (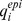 in Equation (4)), changing TF concentration would not change the contributions from other factors to the binding affinity (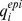). Thus, the analyses above hold for any TF concentrations.

Next, we examined the cooperativity of adjacent TFBSs with simulations. With nearly no epigenomic modifications, a simulated genomic sequence containing two weak TFBSs exhibited a binding affinity larger than that of another sequence containing one weak TFBS (dashed and solid blue curves at epigenomic intensity = 10^−2^, Figure 5B), but smaller than that of a medium-strength TFBS and a strong TFBS (green and red curves at epigenomic intensity = 10^−2^, Figure 5B). As the intensity of an activation mark increased, the binding affinity of the two-weak-TFBS sequence first surpassed that of the medium-strength TFBS and later superseded the strong TFBS to become the sequence with the largest binding strength (Figure 5B). This suggests that in the presence of the epigenome, the binding affinities of different genomic sequences may not always be monotonic. Considering that without cooperativity, the binding affinities of different sequences are strictly monotonic (Figure 5A), these data suggest that epigenomic modifications are not only capable of increasing the binding affinity of each of the two weak TFBSs, but also can increase the cooperativity between the two TFBSs.

Finally, we examined whether the binding affinity of the two weak TFBSs could surpass that of the medium-strength TFBS within the range of typical intensities of epigenomic modifications measured by ChIP-seq experiments. The dashed curve and the green curve crossed at the epigenomic intensity of 10^0.12^ ( = 1.32), corresponding to the enrichment ratio of e^1.32^ ( = 3.74) between the number of sequence reads in the input and the control samples. Because the enrichment ratio of these two numbers is typically between 1 and 40 [53], the change of order of the binding affinities of these two simulated genomic sequences can happen in typical epigenomic conditions.

### Epigenomic boost of weak TFBSs is potentially a regulatory mechanism

With the theoretical understanding that epigenomic modifications can boost the cooperativity of weak TFBSs, we hypothesized that this is a general mechanism of quantitative regulation of gene expression. We explored this hypothesis with tri-methylation of Histone 3 Lysine 4 (H3K4me3) and the transcription factor Oct4, which is essential for maintaining undifferentiation [54], [55] of mES cells (Text S1). Using Oct4 PSWM, we scanned all Oct4 binding regions, which were defined by the peaks in ChIP-seq data in mES cells [33]. We categorized the Oct4 TFBSs into two sets, strong TFBSs (2055 regions, Text S1) and weak TFBSs (1921 regions). The average H3K4me3 intensity on weak-TFBSs was larger than 150% of that on strong-TFBSs (p-value<10^−20^, Figure 5C). The largest difference of H3K4me3 intensities between the two sets appeared at the center of Oct4 binding regions (Position = 0, Figure 5C). This suggests that on Oct4 binding regions throughout the genome, H3K4me3 is more concentrated on those containing only weak sequence motifs. We ruled out promoters as a confounding factor to the association of strong H3K4me3 to weak TFBSs, because weak TFBSs do not preferentially locate in promoters (Chi-square test p-value = 0.907, Table S3, Text S1).

We then asked if these weak-TFBS-only sequences could obtain a larger boost of binding affinity than the other sequences. Our simulation analysis suggested this was the case in theory (Figure 5B). We now test it with the measured epigenomic and TF binding intensities in mES cells. We classified the ChIP-seq peaks into three sets, those only containing strong TFBSs, those containing both strong and weak TFBSs (mixed), and those only containing weak TFBSs. We computed the change in Oct4 binding affinities on these sequence sets from not using H3K4me3 ChIP-seq data to using H3K4me3 ChIP-seq data. The weak-TFBS-only set exhibited a larger increase in binding affinities than the mixed set, which in turn had a larger increase than the strong-TFBS-only set (Figures 5D, S5). These data suggest that the endogenous levels of H3K4me3 in mES cells are sufficient to boost the binding affinity of adjacent weak TFBSs.

Finally, had epigenomic boost of weak TFBSs happened in vivo, the model would be able to better reproduce *in vivo* binding intensities on weak TFBSs. To test this idea, we used DNA sequence and H3K4me3 to predict Oct4 binding regions and compared with ChIP-seq data. We quantified the improvements of the prediction accuracy between with and without considering H3K4me3 data. Applying the model with a stringent threshold on the predicted binding probability, the three sequence groups that harbor strong, mixed, and weak sites showed similar improvements on prediction accuracy (strong-cutoff, Figure 5E). This indicates H3K4me3 helps to improve prediction, but does not specifically show it helps prediction on weak sites. Under two less stringent thresholds of the predicted binding probabilities, the model gained larger increases of prediction accuracy on weak sites and on mixed sites than on strong sites (Medium-cutoff, Weak-cutoff, Figure 5E). These results are consistent with the idea that the model was able to predict the binding intensities more accurately by capturing the epigenomic boost of weak TFBSs. Besides H3K4me3 and Oct4, several other epigenomic marks showed systematically stronger intensities near the weak TFBSs than near the strong TFBSs of other TFs (Table S4). Thus, epigenomic boost of the binding affinity of adjacent weak TFBSs is not only a theoretical possibility, but also can be a wide-spread regulatory mechanism.

### H3K9ac and H3K4me2 may dampen the variation of TF binding across human individuals

Genomic variations including single nucleotide polymorphisms (SNPs) can result in phenotypic variation. Still unknown is how epigenomes modulate the correlation of genotypes and phenotypes among humans. We chose TF binding intensities as a molecular phenotype to study this question.

To study how epigenetic variation can interact with genomic variation, we did three between-individual comparisons across different ethnic groups. We first compared a European (NIGMS catalog ID: GM12878) and a Nigerian (GM18505). We categorized NFκB binding regions with the TFBSs containing SNPs into two sets (all analyses were done with homozygous SNPs, Text S1). The first set had differences in NFκB binding intensities between these two individuals. This set was called Different Sequence Different Binding (DSDB) (Figure S6A). The second SNP-containing set had similar NFκB binding levels in the two individuals, and were termed the Different Sequence No Difference in Binding (DSNDB) set. The first set (DSDB) was consistent with the theory that nucleotide changes in the TFBS should change the binding affinity of this TFBS; however, the second set (DSNDB) appeared to be inconsistent with such a theory. We hypothesized that the epigenetic marks on DSNDB stabilized the binding affinities of these binding sites. In other words, the epigenetic modifications on the TFBSs buffered sequence changes (SNPs) from changing binding intensities.

Theoretically, the difference in binding affinities between two TFBSs is the largest without any epigenetic marks (y-intercept, Figure 5A). When epigenetic modification intensities increase, the binding difference in the two TFBSs decreases (from left to right, Figure 5A). This is true for any two TFBSs of the same TF. Thus, we have derived a theoretical mechanism for the epigenome to attenuate the TF binding differences on SNP-containing TFBSs in two individuals.

**Figure 6:**
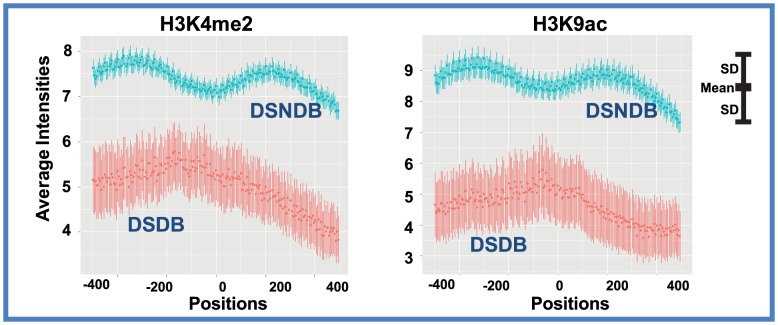
H3K9ac and H3K4me2 dampen personal variation of NFκB binding. The average intensities of H3K9ac and H3K4me2 are higher in DSNDB regions (blue) than in DSDB regions (red). The centers of all NFκB ChIP-seq peaks are superimposed to ‘Position 0’ on the x axis. DSNDB: different sequence no difference in binding. DSDB: different sequence different binding. SD: standard deviation.

We proceeded to examine whether the theoretical mechanism is relevant for transcription factor binding in humans. We first used our model to learn epigenetic marks that help to explain the binding intensities in all SNP-containing TFBSs (Table S5). Four epigenetic marks were identified by the model, which were H3K4me1/2, H3K9ac, and H3K27ac (Figure S7). Among them, H3K4me2 and H3K9ac were identified as marks that better explain the binding intensities in DSNDB sites. If H3K4me2 and H3K9ac were used to attenuate binding differences between two people, there should be higher intensities of H3K4me2 and H3K9ac in DSNDB sites than in DSDB sites. Indeed, the intensities of H3K4me2 and H3K9ac were much higher in DSNDB sites than in DSDB sites (p-values<10^−20^, Figure 6). To assess whether these results were specific to the chosen individuals in our analysis, we did two more comparisons. The second comparison was between a European (GM12878) and a Nigerian (GM19099), and the third comparison was between a European descendant (GM12878) and a Japanese (GM18951). Each comparison identified its own DSDB and DSNDB sites. However, all comparisons found significantly higher H3K4me2 and H3K9ac intensities in DSNDB sites than in DSDB sites (Figure S8). The NFκB binding intensities in DSDB and DSNDB of GM12878 had similar distributions, and therefore are unlikely to contribute to explain the differences of H3K4me2 and H3K9ac intensities in GM12878 (Figure S6B–C). As a control, adding H3K36me3 data to the model did not increase the correlation of model predicted binding intensities to NFκB ChIP-seq data (Figure S7). Accordingly, the difference in H3K36me3 levels between DSDB and DSNDB sets was not clear and not consistent in these comparisons (Figure S8). Finally, we assessed whether inter-individual differences of PSWM matching scores were significantly different in DSDB and DSNDB regions. No significant differences were found in two pairs of individuals (Figure S9, S10), ruling out the possibility that sequence-determined differences in binding energies were more pronounced in either of the two sequence sets. These data suggested a mechanistic explanation to the SNPs in TFBSs that do not produce between-individual differences in TF binding: epigenetic modifications on these TFBSs attenuated the binding differences. We note that not all factors relevant to gene regulation have been considered in this analysis. Other factors including DNase sensitivity and the binding of other TFs could play a role in buffering polymorphism in NFkB binding sites and therefore potential provide alternative explanations.

## Discussion

The overarching tenet of this work is obtaining mechanistic insights from high-throughput genomic data. Towards this goal, we forfeited commonly used “statistical enrichment” methods that look for large overlaps of two or more genomic features. Instead, we developed a biophysical model for the three-way interactions among the genomic sequence, the epigenetic modifications, and TF binding. The model is specified as a physical system, and every model parameter has a biophysical interpretation. This allows the analytical results obtained from this model to have mechanistic interpretations.

Several epigenetic modifications were previously assumed to facilitate or hinder TF binding in a ubiquitous manner. For example, mono-, di-, and tri- methylations on histone lysine 4 (H3K4me1/2/3) were thought to facilitate the binding of any TF. Our data suggested that some TFs tend to preferentially recognize TF-specific epigenomic codes. This implies that rather than ubiquitously synergize or antagonizing TF-DNA binding, some epigenetic marks can specifically interact with some TFs. This is conceivable because the maintenance of epigenetic marks often require histone or DNA modification enzymes to be brought to a genomic sequence by specific transcription factors [56], [57]. In addition, epigenetic modifications are strongly associated with the three-dimensional (3D) architectures of the local chromatin [58]. It is also conceivable that some TFs would have different binding preferences to the same DNA sequence but different 3D chromatin conformations. We showed that epigenetic modifications can boost the cooperativity of adjacent weak TFBSs. Thus, there is a functional advantage of coding a cis-regulatory sequence with a cluster of weak TFBSs rather than one strong binding site. The advantage is that the binding affinity of a cluster of weak TFBSs has a larger tunable range than a strong TFBS, in the presence of the epigenome. Thus, clusters of weak TFBSs offer the epigenome larger ‘controllability’. This may explain why weak TFBSs tend to cluster in the mammalian genomes [59]. Consistently, we estimated that there were 2.3–4.6 weak Oct4 sites per ChIP-seq derived Oct4 binding region. Indeed, H3K4me3 was strongly enriched in Oct4 binding regions that only contained weak TFBSs. Moreover, H3K4me3 generated larger enhancements of binding affinities in the weak-TFBS-only binding regions than in other Oct4 binding regions. Thus, the ‘epigenomic boost’ of TFBS cooperativity can be a functional mechanism in mammalian cells. This provides an alternative view on the evolutionary origin of TFBS clusters, in which the presence of the epigenome was previously ignored [60].

A central question in personalized medicine is how genomic variation generates phenotypic variation. This is a challenging question because genomic variation was only partially correlated with TF-binding variation [61]. In particular, a set of SNPs in TFBSs does not introduce differences to TF binding as predicted by available TF-DNA binding models. Incorporating the epigenome into the TF-DNA binding model, we can now appreciate that some epigenetic marks can buffer genomic changes from generating changes in TF binding intensities. A case in point is that H3K4me2 and H3K9ac attenuate the personal variation of NFκB binding on SNP-containing binding sites in human lymphocytes. These results highlight the importance of considering the epigenome when analyzing the functional consequences of genomic variations.

A limitation of the thermodynamic equilibrium model is that it does not make causal inferences. It does not differentiate whether an epigenomic motif promotes the binding of a TF, or the binding of a TF causes the buildup of an epigenomic motif. It is conceivable that TF binding and epigenomic motif can reinforce each other, in a sequence-dependent or sequence-independent manner. Recent cross-species comparisons reported larger evolutionary changes of TF binding regions [62] than epigenetically modified regions [43]. If we assume during evolution we should see larger changes in the effects than in the causes, then these data are compatible to the hypothesis that epigenetic factors could modulate the binding of specific TFs. Moreover, the model-identified epigenomic mark that have strong interaction with Oct4 binding is H3K4me3, whose intensity is much larger in the Oct4 binding regions that contain only weak TFBSs than in the binding regions that contain strong TFBSs (Figure 5C). If Oct4 binding had been the cause of H3K4 tri-methylation, we would expect H3K4me3 to be stronger on the regions containing strong Oct4 sites. Thus, at least in the case of Oct4 and H3K4me3 interaction, the data disfavor TF binding as the cause of this interaction.

## Methods

### Model assumptions

First, a DNA sequence is associated with a physical state, which is defined by the combination of transcription factors bound to the sequence. When we consider one piece of genomic sequence a time, the physical state of a sequence can be regarded as the physical state of a cell. Second, TF-DNA binding has reached thermodynamic equilibrium, which implies the proportion of cells at each physical state does not change over time. Third, the binding affinity between a TF to any genomic location is a joint effect of multiple TFBSs in the “neighborhood” of this genomic location. Each TFBS has its own binding strength, and they may cooperate. Finally, the intensity of an epigenomic modification in this genomic neighborhood can influence TF binding.

### Model formulation

We model a genomic sequence (S) in a fixed epigenomic context as a physical system. Every TFBS in S can exist in one of the two physical states, occupied or not occupied by a TF. Thus, a sequence containing n TFBSs can exist in any of the total of 2^n^ states (Figure 1A shows the 2^3^ states for a sequence containing 3 TFBSs). We use c to denote a state, and let C to denote all states. There is certain probability associated with every state of the system, denoted as P(c). Such a probabilistic distribution is called a Boltzmann distribution [21].

From the perspective of a particular TF (named A), the event that A is bound to sequence S is equivalent to the union of some of states of S. In the example in Figure 1, the event ‘A is bound’ is the union of States 2, 4, 5, 6, 7, and 8. We call these states the *occupied states* (*O*). Obviously, 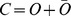. The probability that A is bound to S is 
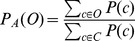
. We introduce the Boltzmann weight, 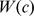, for every state *c*. 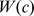 is proportional to 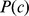 in the way that 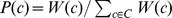. Thus, the probability that A is bound to S is
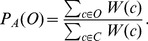
(1)


We model the Boltzmann weight 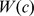 as follows. Two factors contribute to 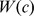. The first factor is the binding affinity between the TF (A) and every TFBS, which is jointly determined by the TFBS and the epigenomic context. We denote this factor as *q^e^* ^***pi***^. The second factor is the cooperativity between TFBSs, denoted as 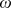. We formulate these thoughts as
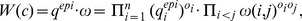
(2)where *i* and *j* are the indices of the TFBSs on S; and *o_i_* is the indicator of whether the *i*th TFBS is occupied (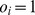, if occupied; 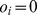, otherwise). This formulation implies that the state with no TFBSs bound (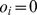 for every *i*) has a Boltzmann weight of 1 (State 1 in Figure 1A). Suppose the *i*th and the *j*th TFBSs are bound by TFs A and B, respectively; 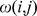 is modeled as
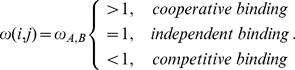
(3)


We then model the binding affinity (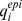) between TF A and the *i* ^th^ TFBS (denoted by *S_i_*). Three factors can contribute: the TF concentration ([*A*]), the preference of the TF to bind onto the binding site sequence *S_i_* (denoted as *K*(*S_i_*)), and the epigenomic influence (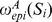). These are modeled as
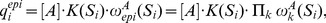
(4)where *k* is an index for each type of epigenomic modification. Here *K*(*S_i_*) is the association constant of the binding site *S_i_*. We note that 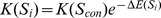, where *S_con_* is the consensus binding site and Δ*E*(*S_i_*) is the extra energy needed to bind onto a non-consensus sequence, which is correlated with the usual matching score between a TFBS (*S_i_*) and the PSWM of the TF. 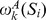 represents the influence of the *k* ^th^ epigenomic modification on the binding intensity on *S_i_*.

We model the TFBS-specific epigenomic influence 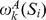 as follows. Let 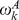 be the overall effect of the *k* ^th^ epigenomic modification to transcription factor A,
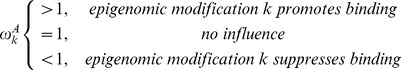
(5)


The TFBS-specific effect 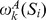 is a joint effect of the overall effect (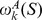) and the intensity of the *k* ^th^ epigenomic modification on *S_i_* (denoted as 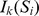). Taking the ChIP-seq data for a histone modification for example, 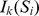 is measured by the ratio of the number of sequencing reads between the experimental sample and the control sample. This study used the number of extended sequencing reads (Text S1) falling on *S_i_*. We model the joint effect as
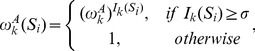
(6)where σ is a threshold determining whether the measured intensity is beyond noise level. We note that 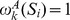 implies either there is no detectable *k* ^th^ modification or the *k* ^th^ modification has no influence to the binding. Figure 1A illustrates how this model works for a sequence with three TFBSs and two partially overlapping epigenomic modifications. We call this model an epigenome-sensitive TF-DNA binding model.

### Making inferences with the model

This model has two major applications. One is to predict the binding intensities of a TF throughout the genome in any cell type. The other application is to learn genomic-location-specific epigenomic influences on TF binding, i.e. 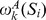. A third and relatively minor application is to learn the cooperativity between TFBSs in different epigenomic contexts. The required inputs are the genome sequence, the PSWM of the TF, and the epigenomic data. Epigenomic data are often generated by ChIP-seq, MeDIP-seq, and other sequencing based experiments. Standard analysis packages, including sequence mapping [63] and mapped reads postprocessing [42] can process each dataset into a genome-wide distribution of the intensity of an epigenomic modification. Our model takes such a distribution as an input through 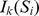, the intensity of the *k*th epigenomic modification on *S_i_*.

### Statistical learning with the model

The model has two sets of models parameters, which are the cooperativity between TFBSs (*ω_A,B_*) and the influence of each epigenomic modification (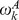). To train these model parameters, four inputs are required. These include the genome sequence, the PSWM of the TF, the epigenomic data (ChIP-seq and other forms), and the ChIP-seq data of the TF of interest. Let *I*(*A*) be the genome-wide distribution of binding intensities of transcription factor A. For example, if we segregate the human genome into 6 million 500 bp long windows, then *I*(*A*) is a vector of 6 million elements. Each element represents the ChIP-seq measured binding intensity in the corresponding window. Following previous notations, we use *P_A_*(*O*) in equation (1) to denote the model predicted binding probability of A in every window. We propose to learn the model parameters by maximizing the following target function
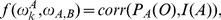
(7)where *corr*(.) is the Pearson Correlation, and *P_A_*(*O*) is a function of 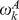 and *ω_A,B_*.

### Computational strategy

We implemented a maximization strategy to maximize *f*(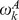, *ω_A,B_*). The analytical form of *P*(*A*) can be explicitly expressed with a dynamic programming algorithm [28]. We maximize it by the Quasi-Newton Method (a.k.a. BFGS algorithm) provided in the GNU Scientific Library [25], [64]. We start with random initial parameters and repeat it 500 times to avoid local minima. In applications where the cooperativity among TFBSs is not of interest, we propose to ignore the cooperativity term (set *ω_A,B_* = 1) and only maximize with respect to 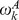.

### Identification of TF-specific epigenomic interactions

We identify an epigenomic modification *k* as associated with the binding of TF *A* when 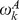
≫1 (positive) or 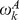
≪1 (negative). To test for the null hypothesis that 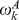 = 1, we shuffle the intensities of epigenomic modification *k* on the genome to obtain 200 random epigenomic profiles. We subsequently compute 200 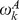 values from the shuffled data and use them as the empirical null distribution. For each epigenomic modification *k*, we test 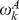 = 1 using the empirical null distribution and reject the null hypothesis using a multiple-hypothesis-adjusted p-value [65] (Figure 1B).

## Supporting Information

Figure S1
**Comparison of different metrics of prediction power.** Model predicted binding intensities are correlated to ChIP-seq reported binding intensities (y axis). The model predictions were based on sequence data alone (Control-1), sequence data plus randomized epigenomic data (Control-2), or sequence data plus one epigenomic mark (other columns). Results on training data (shaded bars) and testing data (hollow bars) using Spearman correlation (red bars) and Pearson correlation (blue bars) are plotted. The model inferred influence of each epigenomic mark to Nanog binding ( in Equation (5)) is given in the brackets following each mark.(EPS)

Figure S2
**Comparison of window sizes on model predictions.** Model predicted binding intensities are correlated to ChIP-seq reported binding intensities (y axis). The model predictions were based on sequence data alone (Control-1), sequence data plus randomized epigenomic data (Control-2), or sequence data plus one epigenomic mark (other columns). Results on training data (shaded bars) and testing data (hollow bars) with the window sizes of 350 bp (red bars) and 500 bp (blue bars) are plotted. The model inferred influence of each epigenomic mark to Nanog binding (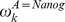 in Equation (5)) is given in the brackets following each mark.(EPS)

Figure S3 **Comparing model predictions with different sequence motifs.** Using the Nanog dataset, we compared model predictions in four scenarios. In each scenario, the model predictions were correlated to ChIP-seq measured Nanog binding intensities (y axis). These scenarios are: 1, simple correlation between epigenomic data and binding data without using the model (solid pink bars); 2, using each epigenomic mark with all the (214) PSWMs from the JASPAR database (hollow pink bars: the mean of the 214 correlations, error bar: standard deviation of the mean); 3, using each epigenomic modification with the *in vivo* Nanog motif (solid blue bars); 4, using each epigenomic modification with the *in vitro* Nanog motif (hollow blue bars).
(EPS)

Figure S4 **Differences of the predicted TF binding regions from the in vivo and the in vitro Nanog motifs.** The in vivo and in vitro motifs with epigenetic data (Here H3K4me1 as an example) were used to predict TF binding regions (BRs). The prediction based on the model returning a binding probability score within [0, 1], and the TF BRs were called by applying a threshold on this probability score. The numbers of TF BRs were called with a high threshold (A, B) and a low threshold (C, D) in both training data (A, C) and testing data (B, D). The numbers of true positive TF BRs (verified by ChIP-seq) are given outside of the parentheses. The total numbers of predicted TF BRs, including both true positives and true negatives are given inside the parentheses.
(EPS)

Figure S5 **Comparison of different cutoffs on calling strong and weak binding sites.** The difference of model-predicted binding probabilities with and without the epigenome (y axis) is larger in weak-TFBS-only regions (right column) than in the regions containing both strong and weak sites (mixed, middle column), which in turn is larger than in the strong-TFBS-only regions (left column). The thresholds for calling strong sites and weak sites are *K*(*S_con_*) – 3.5 and *K*(*S_con_*) – 7.0, respectively, where *K*(*S_con_*) is the consensus score. These thresholds are different from those used in Figure 5D.
(EPS)

Figure S6 **Variations of the strengths of NFκB binding regions.** (A) The inter-individual variation of the strengths of NFκB binding regions are quantified by Difference Ratio ( *DR*, y axis), which is defined as DR = |*I*(*S_i_*) - *I*(*S_j_*)|/min( *I*(*S_i_*), *I*(*S_j_*)), where *I*(*S_i_*) and *I*(*S_j_*) are the binding strengths of sequences *S_i_* and *S_j_* in individuals *i* and *j* measured by ChIP-seq experiments. The mean (each bar) and standard error (error bars) of *DR*s in DSDB (left) and DSNDB sequence sets (right) are shown. The distribution of GM12878 NFκB binding in DSDB (left) and DSNDB (right) sequence sets, where DSDB and DSNDB were identified from the comparison of GM12878 and GM18505 (B) and from the comparison of GM12878 and GM12892 (C). CEU: Northern and western Europe. YRI: Nigeria. SE: standard error.
(EPS)

Figure S7 **Interactions of NFκB and epigenomic marks.** The Pearson correlation between model-predicted and ChIP-seq measured binding intensities (x axis) is used to identify the epigenomic marks interacting with NFκB. The genomic sequence and ChIP-seq data of GM12878 were used to fit the model. DSDB and DSNDB sequences were identified from comparing sequence and epigenomic data of GM12878 and GM18505. The results from four-fold cross validations are shown. Shaded bars: training data. Hollow bars: testing data. Length of each bar: the average value from four-fold cross validations. A total of 200 randomized epi- datasets were used as controls (Control). Four-fold cross validations were performed on each randomized dataset. The mean correlation from these four-fold cross validations of 200 random datasets is represented by the length of each Control bar. Error bars: standard deviations of the mean. The epigenomic marks that significantly increase the Pearson correlation from the control experiments are identified (*, p-value<0.01).
(EPS)

Figure S8 **H3K9ac and H3K4me2 are associated with small variation of NFκB binding.** Two other comparisons (GM12878 vs. GM19099 and GM12878 vs. GM18951) confirm that the average intensities of H3K9ac and H3K4me2 are higher in DSNDB regions (blue) than in DSDB regions (red). As a control, no reproducible differences between DSNDB and DSDB regions are found for H3K36me3. The centers of all NFκB ChIP-seq peaks are superimposed to ‘Position 0’ on the x axis. DSNDB: different sequence no difference in binding. DSDB: different sequence different binding. SD: standard deviation. CEU: Northern and western Europe. YRI: Nigeria. JPT: Japan.
(EPS)

Figure S9 **Variations of binding energies of the TFBSs with SNPs.** (**A**)The inter-individual variation of the TFBS binding energies was determined by PSWM scores. For individuals *i* and *j* with a SNP in a NFκB binding region *S_k_*, the absolute difference of sequence-determined binding energies is defined as |e_i_(*S_k_*) – e_j_(*S_k_*)|, where e_i_(*S_k_*) and e_j_(*S_k_*) are the PSWM scores of TFBS sequence *S_k_* in individuals *i* and *j*. The mean (each bar) and standard error (error bar) in CEU (left) and CEUYRI (right) are shown. The distribution of variations of binding energies in (B) CEU and (C) CEUYRI comparison.
(PDF)

Figure S10 **Distribution of PSWM matching scores in DSDB and DSNDB regions.** (A) CEU (B) CEUYRI comparison.
(PDF)

Table S1 **(A) Biophysical models of TF-DNA binding.** (B) Machine learning models to incorporate epigenomic information on TF binding.
(DOCX)

Table S2
**Comparison of model performances with and without epigenomic data.** Transcription factor binding and epigenomic data in mES cells were used as inputs. Model-inferred interacting epigenomic marks of each transcription factor (row) are reported (2^nd^ column). Model performances were evaluated with Pearson correlation using both sequence data and epigenomic data (3^rd^ column) and using sequence data alone (4^th^ column). The improvement was quantified as the difference of the correlations divided by the correlation without epigenomic data (5^th^ column). 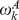: the overall effect of the *k* ^th^ epigenomic modification to transcription factor A, as defined in Equation (5).(DOCX)

Table S3 **Lack of association between weak TFBSs and promoters.** The distribution of strong and weak TFBSs in promoters and other regions are summarized. Chi-square test p-value = 0.907.
(DOCX)

Table S4 **Epigenomic marks with greater intensities near weak TFBS.** For each TF (row) and each epigenomic mark (column), we tested whether the ChIP-seq signals of this epigenomic mark were significantly different near weak TFBSs than those near strong TFBSs. Significant differences of epigenomic intensities were marked with “v”.
(DOCX)

Table S5 **Distribution of SNP-containing NFκB binding sites.** The numbers of NFκB binding sites that contain polymorphic nucleotides between two individuals are summarized. Depending on whether these polymorphic nucleotides generated differences in NFκB binding intensities, these SNP-containing binding sites are separately counted. DSDB: Different Sequence Different Binding. DSNDB: Different Sequence No Difference in Binding. CEU: A person from northern or western Europe. YRI: A person from Nigeria.
(DOCX)

Text S1 **Supplementary methods.**
(DOCX)
