# Supplementary figures and images for: Understanding Variation in Transcription Factor Binding by Modeling Transcription Factor Genome-Epigenome Interactions
Source: PLoS Comput Biol. 2013 Dec 5;9(12):e1003367. doi: 10.1371/journal.pcbi.1003367 (PMC3854512; doi:10.1371/journal.pcbi.1003367)

**A**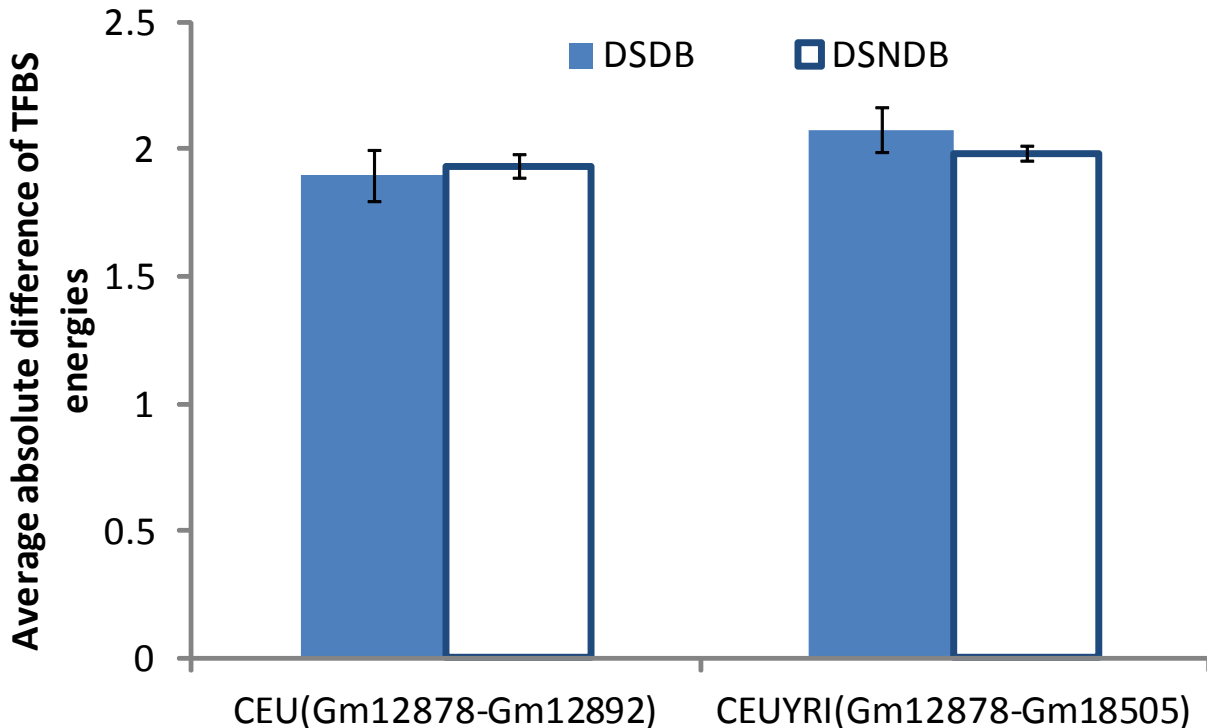**B****CEU(Gm12878-Gm12892)**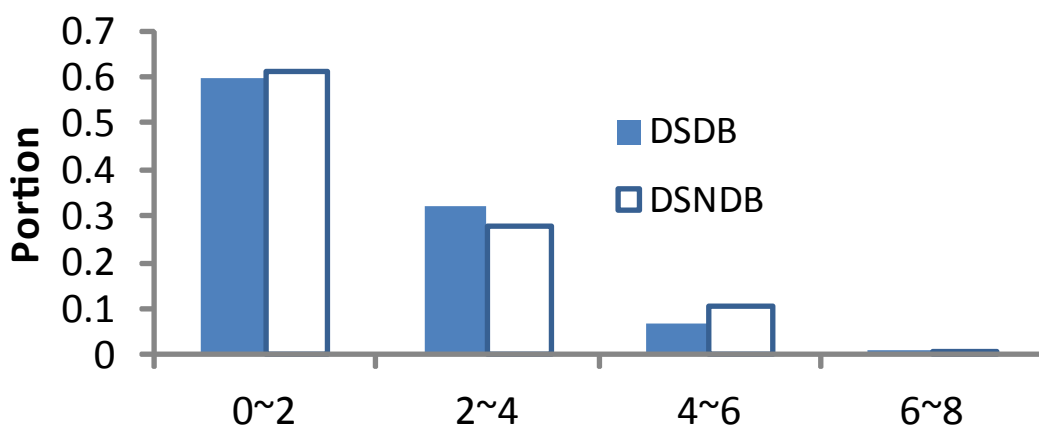**C****CEUYRI (Gm12878-Gm18505)**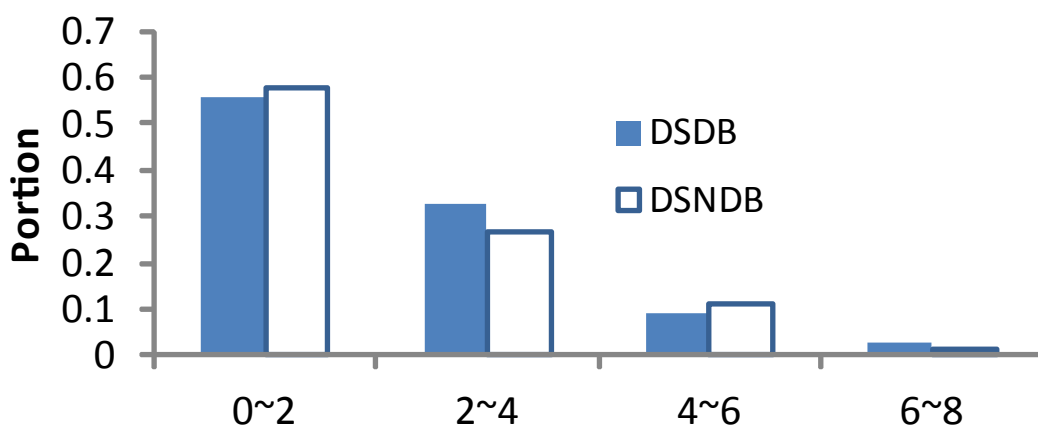

Distribution of average absolute differences of TFBS energies

Supplement: Figure S9 — Variations of binding energies of the TFBSs with SNPs. (A)The inter-individual variation of the TFBS binding energies was determined by PSWM scores. For individuals i and j with a SNP in a NFκB binding region Sk, the absolute difference of sequence-determined binding energies is defined as |ei(Sk) – ej(Sk)|, where ei(Sk) and ej(Sk) are the PSWM scores of TFBS sequence Sk in individuals i and j. The mean (each bar) and standard error (error bar) in CEU (left) and CEUYRI (right) are shown. The distribution of variations of binding energies in (B) CEU and (C) CEUYRI comparison. (PDF) [file pcbi.1003367.s009.pdf]

### A. CEU(Gm12878-Gm12892)

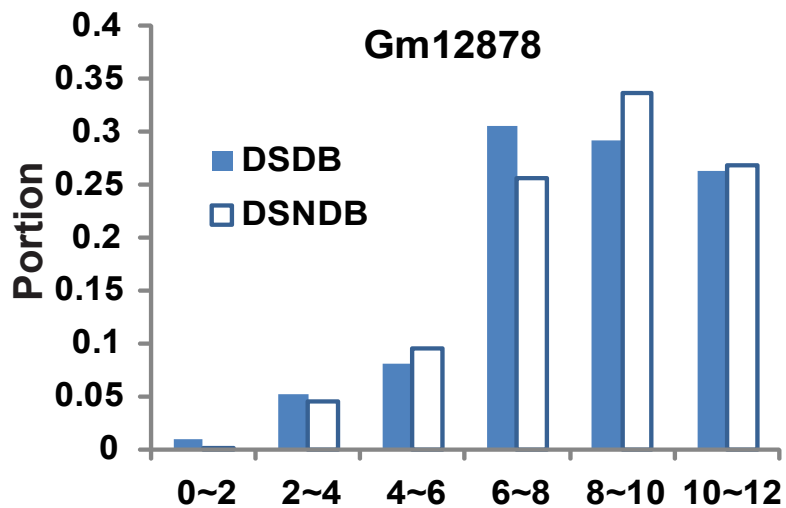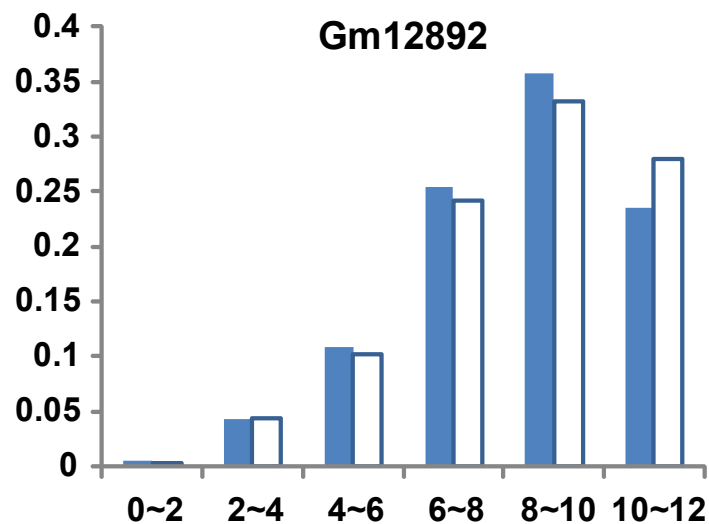

### B. CEUYRI(Gm12878-Gm18505)

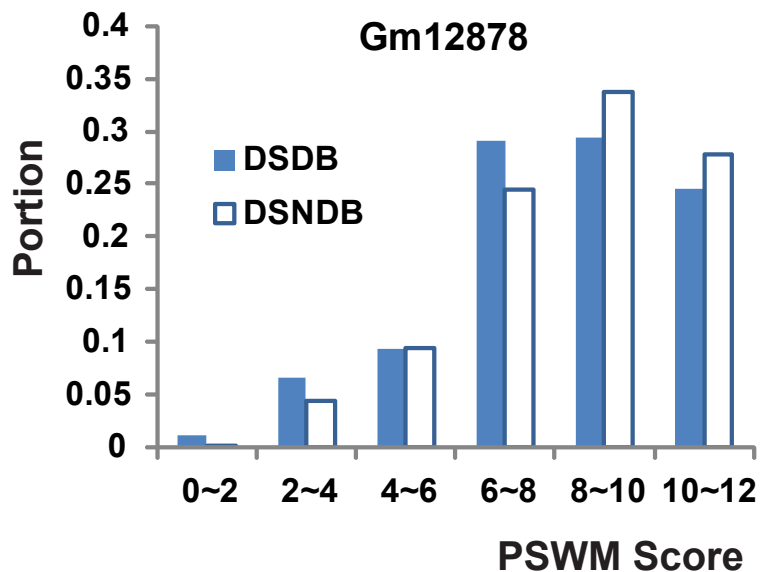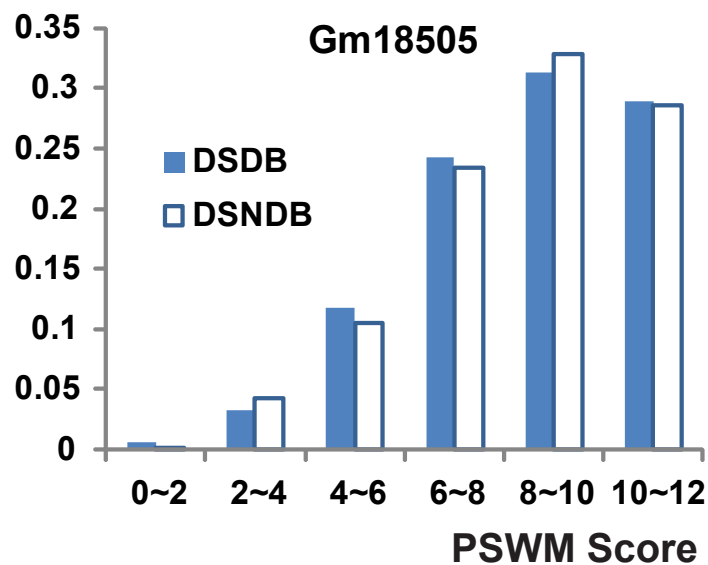

Supplement: Figure S10 — Distribution of PSWM matching scores in DSDB and DSNDB regions. (A) CEU (B) CEUYRI comparison. (PDF) [file pcbi.1003367.s010.pdf]
