# Supplementary material for: Understanding Variation in Transcription Factor Binding by Modeling Transcription Factor Genome-Epigenome Interactions
Source: PLoS Comput Biol. 2013 Dec 5;9(12):e1003367. doi: 10.1371/journal.pcbi.1003367 (PMC3854512; doi:10.1371/journal.pcbi.1003367)
Supplement: Table S1 — (A) Biophysical models of TF-DNA binding. (B) Machine learning models to incorporate epigenomic information on TF binding. (DOCX) [file pcbi.1003367.s011.docx]

­Table S1. (A) Biophysical models of TF-DNA binding. (B) Machine learning models to incorporate epigenomic information on TF binding.

**A. Biophysical models**

| Methods | Model assumptions | | |  | Applications | |
| --- | --- | --- | --- | --- | --- | --- |
|  | **Var. TFBS affinities** | **TF coop.** | **Epi- data** |  | **HT data** | **Species** |
| Shea et al [[1](#_ENREF_1)]  Bulcher et al [[2](#_ENREF_2)] | N | P^1^ | N |  | N | Bacteria |
| Janssens et al. [[3](#_ENREF_3)] | Y | N | N |  | N | Drosophila |
| Segal et al. [[4](#_ENREF_4)] | Y | Y | N |  | N | Drosophila |
| Gertz et al. [[5](#_ENREF_5)] | N | P^1^ | N |  | N | Synthetic promoters in yeast |
| He et al. [[6](#_ENREF_6)] | Y | Y | N |  | Y | Drosophila and mice |
| Fakhouri et al. [[7](#_ENREF_7)] | Y | Y | N |  | N | Drosophila |
| Raveh-Sadka et al. [[8](#_ENREF_8)] | Y | Y | P^2^ |  | N | Yeast |
| Mirny [[9](#_ENREF_9)] | N | Y | P^2^ |  | N | Simulation data |
| This work | Y | Y | Y |  | Y | Humans and mice |
| Y: Yes. N: No. P: Partial consideration.  P^1^: TF-TF interactions are modeled as invariant to any participating TFs.  P^2^ : Nucleosome positions are considered but histone and DNA modifications are not modeled.  Var. TFBS affinities: TFBS affinities are modeled as specific to the TFBS sequence.  TF coop. : Cooperativity between TFBSs.  Epi- data: Modeling epigenomic environment.  HT data: Applicability to analyzing high-throughput genomic data. | | | | | | |

**B. Machine learning methods**

| Methods |  | Model assumptions | | | HT data used | |
| --- | --- | --- | --- | --- | --- | --- |
|  |  | **Var. TFBS affinities** | **TF coop.** | **Epi- data** |  |  |
| Ernst et al. [[10](#_ENREF_10)] |  | Y | P^2^ | P^1^ |  | Y |
| Boyle et al. [[11](#_ENREF_11)] |  | Y | N | P^1^ |  | Y |
| Arvey et al. [[12](#_ENREF_12)] |  | Y | N | P^3^ |  | Y |
| P^1^ : Open chromatin regions are considered but specific histone and DNA modifications are not modeled.  P^2^: TF-TF interactions are modeled as invariant to the specific participating TFs.  P^3^: Both open chromatin and histone modifications are considered but only the former contributed to the predictors of the final model. | | | | | | |

1. Shea MA, Ackers GK (1985) The OR control system of bacteriophage lambda. A physical-chemical model for gene regulation. J Mol Biol 181: 211-230.

2. Buchler NE, Gerland U, Hwa T (2003) On schemes of combinatorial transcription logic. Proc Natl Acad Sci U S A 100: 5136-5141.

3. Janssens H, Hou S, Jaeger J, Kim AR, Myasnikova E, et al. (2006) Quantitative and predictive model of transcriptional control of the Drosophila melanogaster even skipped gene. Nat Genet 38: 1159-1165.

4. Segal E, Raveh-Sadka T, Schroeder M, Unnerstall U, Gaul U (2008) Predicting expression patterns from regulatory sequence in Drosophila segmentation. Nature 451: 535-540.

5. Gertz J, Siggia ED, Cohen BA (2009) Analysis of combinatorial cis-regulation in synthetic and genomic promoters. Nature 457: 215-218.

6. He X, Chen CC, Hong F, Fang F, Sinha S, et al. (2009) A biophysical model for analysis of transcription factor interaction and binding site arrangement from genome-wide binding data. PLoS One 4: e8155.

7. Fakhouri WD, Ay A, Sayal R, Dresch J, Dayringer E, et al. (2010) Deciphering a transcriptional regulatory code: modeling short-range repression in the Drosophila embryo. Mol Syst Biol 6: 341.

8. Raveh-Sadka T, Levo M, Segal E (2009) Incorporating nucleosomes into thermodynamic models of transcription regulation. Genome Res 19: 1480-1496.

9. Mirny LA (2010) Nucleosome-mediated cooperativity between transcription factors. Proc Natl Acad Sci U S A 107: 22534-22539.

10. Ernst J, Plasterer HL, Simon I, Bar-Joseph Z (2010) Integrating multiple evidence sources to predict transcription factor binding in the human genome. Genome Research 20: 526-536.

11. Boyle AP, Song L, Lee B-K, London D, Keefe D, et al. (2011) High-resolution genome-wide in vivo footprinting of diverse transcription factors in human cells. Genome Research 21: 456-464.

12. Arvey A, Agius P, Noble WS, Leslie C (2012) Sequence and chromatin determinants of cell-type–specific transcription factor binding. Genome Research 22: 1723-1734.
