# Supplementary material for: Understanding Variation in Transcription Factor Binding by Modeling Transcription Factor Genome-Epigenome Interactions
Source: PLoS Comput Biol. 2013 Dec 5;9(12):e1003367. doi: 10.1371/journal.pcbi.1003367 (PMC3854512; doi:10.1371/journal.pcbi.1003367)
Supplement: Table S2 — Comparison of model performances with and without epigenomic data. Transcription factor binding and epigenomic data in mES cells were used as inputs. Model-inferred interacting epigenomic marks of each transcription factor (row) are reported (2nd column). Model performances were evaluated with Pearson correlation using both sequence data and epigenomic data (3rd column) and using sequence data alone (4th column). The improvement was quantified as the difference of the correlations divided by the correlation without epigenomic data (5th column). : the overall effect of the k th epigenomic modification to transcription factor A, as defined in Equation (5). (DOCX) [file pcbi.1003367.s012.docx]

Table S2. Comparison of model performances with and without epigenomic data. Transcription factor binding and epigenomic data in mES cells were used as inputs. Model-inferred interacting epigenomic marks of each transcription factor (row) are reported (2^nd^ column). Model performances were evaluated with Pearson correlation using both sequence data and epigenomic data (3^rd^ column) and using sequence data alone (4^th^ column) in the testing dataset. The improvement was quantified as the difference of the correlations divided by the correlation without epigenomic data (5^th^ column). $\omega_{k}^{A}$: the overall effect of the $k$^th^ epigenomic modification to transcription factor A, as defined in Equation (5).

| **Transcription factor** | **Interacting epigenomic marks**  **(**$\boldsymbol{\omega}_{\boldsymbol{k}}^{\boldsymbol{A}}$**, training/testing p-value)** | **Correlation, with epi- marks** | **Correlation, without epi-marks** | **Improvement** |
| --- | --- | --- | --- | --- |
| **cMyc** | H3K4me3(1.78,0.0049/0.0049), H3K9me3(0.06, 0.0049/0.0049), H3K36me3(0.1, 0.0049/0.009), 5-mC(MRE)(1.82, 0.0049/0.0049), 5-mC(MeDIP)(0.06, 0.0049/0.0049), 5-hmC(0.06, 0.0049/0.0049), H2AZ(0.06, 0.0049/0.0049), H3K27ac(2.53, 0.0049/0.0049) | 0.77 | 0.64 | 20.3% |
| **Nanog** | H3K4me1(5.25, 0.0049/0.0049), H3K4me3(11.75, 0.0049/0.0049), 5-mC(MRE)(2.92, 0.0149/0.05), 5-mC(MeDIP)(0.06, 0.005/0.0049), H3K27ac(9.90, 0.0049/0.0049) | 0.63 | 0.21 | 200% |
| **nMyc** | H3K4me3(1.49, 0.0149/0.0049), H3K9me3(0.16, 0.0348/0.0049), 5-mC(MRE)(15.8, 0.0149/0.0049), 5-mC(MeDIP)(0.06, 0.0149/0.0049), 5-hmC(0.06, 0.0049/0.0049), H2AZ(0.06, 0.0049/0.0049), H3K27ac(1.80, 0.0049/0.0049) | 0.78 | 0.74 | 5.41% |
| **Oct4** | H3K4me3(1.67, 0.0049/0.0049), 5-mC(MeDIP)(0.06, 0.019/0.054), H3K27ac(2.15, 0.0049/0.0049) | 0.57 | 0.43 | 32.6% |
| **Sox2** | H3K4me1(2.20, 0.0049/0.0049), H3K4me3(2.57, 0.0049/0.0049), 5-mC(MeDIP)(0.06, 0.009/0.029), H3K27ac(4.21, 0.0049/0.0049) | 0.67 | 0.53 | 26.4% |
| **STAT3** | H3K4me1(1.40, 0.05/0.0049), H3K4me3(1.94, 0.0049/0.019), 5-mC(MeDIP)(0.06,0.019/0.009), H3K27ac(2.41, 0.0049/0.0049) | 0.59 | 0.49 | 20.4% |
| **Esrrb** | No significant marks | N/A | 0.81 | N/A |
| **Klf4** | H3K27ac(2.38, 0.0049/0.0049) | 0.82 | 0.80 | 2.5% |
| **Zfx** | H3K27ac(2.19, 0.0049/0.0049) | 0.84 | 0.82 | 2.43% |
