# Supplementary material for: Understanding Variation in Transcription Factor Binding by Modeling Transcription Factor Genome-Epigenome Interactions
Source: PLoS Comput Biol. 2013 Dec 5;9(12):e1003367. doi: 10.1371/journal.pcbi.1003367 (PMC3854512; doi:10.1371/journal.pcbi.1003367)
Supplement: Table S3 — Lack of association between weak TFBSs and promoters. The distribution of strong and weak TFBSs in promoters and other regions are summarized. Chi-square test p-value = 0.907. (DOCX) [file pcbi.1003367.s013.docx]

Table S3. Lack of association between weak TFBSs and promoters. The distribution of strong and weak TFBSs in promoters and other regions are summarized. Chi-square test p-value = 0.907.

|  | Strong TFBSs | Weak TFBSs | Total |
| --- | --- | --- | --- |
| Promoters | 353 | 2004 | 2357 |
| Other regions | 1360 | 6578 | 7938 |
| Total | 1713 | 8582 | 10295 |
