# Supplementary material for: Understanding Variation in Transcription Factor Binding by Modeling Transcription Factor Genome-Epigenome Interactions
Source: PLoS Comput Biol. 2013 Dec 5;9(12):e1003367. doi: 10.1371/journal.pcbi.1003367 (PMC3854512; doi:10.1371/journal.pcbi.1003367)
Supplement: Table S4 — Epigenomic marks with greater intensities near weak TFBS. For each TF (row) and each epigenomic mark (column), we tested whether the ChIP-seq signals of this epigenomic mark were significantly different near weak TFBSs than those near strong TFBSs. Significant differences of epigenomic intensities were marked with “v”. (DOCX) [file pcbi.1003367.s014.docx]

Table S4. Epigenomic marks with greater intensities near weak TFBS. For each TF (row) and each epigenomic mark (column), we tested whether the ChIP-seq signals of this epigenomic mark were significantly different near weak TFBSs than those near strong TFBSs. Significant differences of epigenomic intensities by a permutation test were marked with “v”.

|  | H3K4me1 | H3K4me3 | H3K9me3 | H2AZ | H3K27ac | H3K27me3 | H3K36me3 | MRE |
| --- | --- | --- | --- | --- | --- | --- | --- | --- |
| cMyc |  | v |  |  |  |  |  |  |
| Esrrb |  | v |  |  | v | v |  | v |
| Klf4 | v | v |  | v | v | v | v | v |
| Nanog |  | v |  |  | v |  |  | v |
| nMyc |  | v |  |  |  |  |  | v |
| Oct4 | v | v |  | v | v | v |  | v |
| Sox2 | v | v |  | v | v | v |  |  |
| STAT3 |  | v | v |  |  |  | v |  |
| Zfx |  |  |  | v | v |  |  |  |
