# Supplementary material for: Understanding Variation in Transcription Factor Binding by Modeling Transcription Factor Genome-Epigenome Interactions
Source: PLoS Comput Biol. 2013 Dec 5;9(12):e1003367. doi: 10.1371/journal.pcbi.1003367 (PMC3854512; doi:10.1371/journal.pcbi.1003367)
Supplement: Table S5 — Distribution of SNP-containing NFκB binding sites. The numbers of NFκB binding sites that contain polymorphic nucleotides between two individuals are summarized. Depending on whether these polymorphic nucleotides generated differences in NFκB binding intensities, these SNP-containing binding sites are separately counted. DSDB: Different Sequence Different Binding. DSNDB: Different Sequence No Difference in Binding. CEU: A person from northern or western Europe. YRI: A person from Nigeria. (DOCX) [file pcbi.1003367.s015.docx]

Table S5. Distribution of SNP-containing NFκB binding sites. The numbers of NFκB binding sites that contain polymorphic nucleotides between two individuals are summarized. Depending on whether these polymorphic nucleotides generated differences in NFκB binding intensities, these SNP-containing binding sites are separately counted. DSDB: Different Sequence Different Binding. DSNDB: Different Sequence No Difference in Binding. CEU: A person from northern or western Europe. YRI: A person from Nigeria.

|  | CEU (GM12878) vs. CEU (GM12892) | CEU (GM12878) vs. YRI (GM18505) |
| --- | --- | --- |
| DSDB | 60 | 91 |
| DSNDB | 360 | 1035 |
