# Supplementary material for: Understanding Variation in Transcription Factor Binding by Modeling Transcription Factor Genome-Epigenome Interactions
Source: PLoS Comput Biol. 2013 Dec 5;9(12):e1003367. doi: 10.1371/journal.pcbi.1003367 (PMC3854512; doi:10.1371/journal.pcbi.1003367)
Supplement: Text S1 — Supplementary methods. (DOCX) [file pcbi.1003367.s016.docx]

###### **Supplementary methods**

###### Analysis of genome-wide distribution of 5-methylcytosine, 5-hydromethylcytosine, H2A.Z and H3K27ac in mouse embryonic stem cells.

We used a 200bp window to slide along the whole mouse genome. For an epigenomic (epi-) mark, we counted the number of the reads that fall on each window. The read count of a window was then normalized against the number of 10 million mapped reads of the epi- mark. Note that all reads were extended to 200bps towards 3' end. Then we used a percentage threshold (5%) on the read count distribution to determine which windows were marked by an epi- mark.

###### Simulation study.

To better understand the nature of our quantitative model for transcription factor (TF)-DNA binding in a given epigenomic context, we explored the dynamic aspects of the model framework. The computation depends on the TF-specific constant (i.e. [*TF*]．*K*(*S_con_*), see model formulation section in the main text), the site strength, the effect of the epi- mark on TF-DNA binding, and the intensity of the epi- mark. Here we assigned the parameters plausible but arbitrary values. The TF-specific constant was set to be 2.0, learned by our model from Nanog data in mouse embryonic stem cells. The strong, middle, and weak site strengths were set to be 0.9, 0.6, and 0.3, respectively. The effect terms of an epi- mark for promoting and suppressing TF-DNA binding were set to be 1.5 and 0.5, respectively. The intensity of the epi- mark was set to 2. Unless otherwise stated, we used these parameter set in the simulation study.

###### Exploration of epigenomic effects on weak binding sites.

We identified the TF binding sites (TFBSs) along with their binding energy values from the peak regions identified by chromatin immunoprecipitation followed by sequencing (ChIP-seq) experiment. We then expanded each TFBS into a 200 bp region with the center of the TFBS unchanged. We used an arbitrary energy cutoff (the position-specific weight matrix score of the TFBS, i.e. the log-likelihood score of the TFBS, is bigger than the consensus-motif log-likelihood score – 2.5) to separate TFBSs into two groups: strong sites and weak sites. For each group, we calculated the average values and the standard deviation values of the epi- intensities along each position of the 200bp region. To further explore the epi- effects on weak sites in our model, we separated those binding peak regions from the ChIP-seq experiment into three different groups based on the occurrences of strong/weak sites: strong sites only, mixed (strong and weak sites), and weak sites only. We applied our model with and without epi- data on those three different groups to compare the differences of their binding affinities.

The error bars in Figure 5E are standard deviations derived from a bootstrapping 90% of the input data for 10 times for each dataset.

###### Association test between weak binding sites and promoter regions

We considered TFBSs within upstream/downstream 5,000 bps from TSS to be located in promoter regions. We then used a Chi-square test to examine the association between weak/strong sites and promoter/non-promoter regions.

###### Comparison of epigenomic variations and TF binding variations.

To test the hypothesis that the individual differences in TF binding are greater than the differences in epigenome, we used published nuclear factor κB (NFκB) binding data [[1](#_ENREF_1)] and DNaseI hypersensitivity data [[2](#_ENREF_2)]. We chose those NFκB binding regions with single-nucleotide polymorphisms (SNPs) located on NFκB binding sites (SNP-TFBSs) as the regions of our interest. For each region, we computed the average intensities of NFκB binding and DNaseI hypersensitivity in each individual. We then calculated the average absolute values of the differences across individual samples in both NFκB binding and DNaseI hypersensitivity intensities. The average absolute values of the differences shown in Figure 6 were proportional to the values from the individual GM12878.

###### Exploration of epigenomic effects on individual sequence variation analysis.

We separated the NFκB peak regions with SNP-TFBSs into two categories: Different Sequences with significantly Different Binding (DSDB) and Different Sequences with No significantly Different Binding (DSNDB), based on whether those peak regions show significant binding differences between individual sample comparisons [[1](#_ENREF_1)] . We applied our epi- model on DSDB and DSNDB data across samples using four-fold cross-validation to learn their specific significant epi- partners. To investigate the influences of epi- partners on SNPs binding sites, we calculated the average epi- intensities as well as the standard deviations of the epi- intensities around 1000bp sequences with the center of those binding regions unchanged in the DSDB and DSNDB sets.

### Data Collection and Preprocessing

###### Mouse embryonic stem cell data.

We studied ChIP-seq data on nine TFs active in mouse embryonic stem (mES) cells [[3](#_ENREF_3)]: cMyc, Esrrb, Klf4, Nanog, nMyc, Oct4, Sox2, STAT3 and Zfx. The reported peak regions previously identified on mouse mm8 genome were converted onto mouse mm9 genome by liftOver from UCSC genome browser [[4](#_ENREF_4)].

Histone modification ChIP-seq data in mouse ESCs, H3K4me1 (GEO:GSM281695), H3K4me3 (GEO:GSM367018), H3K9me3 (GEO:GSM307621), H3K27me3 (GEO:GSM397410) and H3K36me3 (GEO:GSM307620) were collected. Together with our data, including 5-mC, 5-hmC, H2A.Z and H3K27ac data, all epi- data mapped onto mouse mm9 genome by bowtie [[5](#_ENREF_5)] with the constraints that only one mismatch was allowed and mapped reads should be uniquely mapped to the genome.

###### Mouse adipose cell data.

We collected a set of histone modification ChIP-seq data in mouse adipose cell as control data. H3K4me1 (GEO:GSM535743), H3K4me3 (GEO:GSM535741), H3K27me3 (GEO:GSM535745) and H3K36me3 (GEO:GSM535746) were obtained and mapped onto mouse mm9 genome by bowtie [[5](#_ENREF_5)] with the same mapping settings.

###### Human lymphocyte cell data.

We collected NFκB ChIP-seq binding data in three individuals [[1](#_ENREF_1)], including GM12878, GM12892 and GM18505. We obtained individual SNP data from April 2009 release of the 1000 Genome project [[6](#_ENREF_6)]. We then collected DNaseI hypersensitivity data of six individuals, including GM12878, GM12892, GM12891, GM19238, GM19239 and GM19240.

The epigenomic data in human lymphocyte cells were available only in GM12878 [[7](#_ENREF_7)]. We collected the following epi- mark data: H3K4me1, H3K4me2, H3K4me3, H3K9ac, H3K27ac, H3K27me3, H3K36me3 and H4K20me1 (GEO:GSE26386). All were mapped onto human hg18 genome by bowtie [[5](#_ENREF_5)] with the above same mapping settings.

###### TF binding data preprocessing and generation of training and testing datasets.

For each ChIP-seq dataset in mES cells, we identified the peak regions and extracted the surrounding sequences, defined as 250bp upstream/downstream of the centers of the peaks as our sequence data. Since the data only presented those regions with strong binding signals, we added an equal number of sequences that did not show significant binding by randomly chosen from the genome. The binding intensities for those randomly unbound sequences are generally unknown. Thus, we used the value below the lowest binding affinity (as 1) among all bound sequences. In general, we only need to use a subset of ChIP-seq data for training the model, while the rest of the data could be used as testing data. In our experiments, we used 1000 sequences, including 500 for positive and 500 for negative sets, as our training dataset. The construction of testing data was in the same fashion, 500 bound regions and 500 random unbound regions.

For human lymphocyte cell data, we extracted the surrounding 1000bp sequences centered on the centers of those peak regions and replaced the sequences with individual SNPs as individual sequence files. In the comparison between GM12878 and GM18505, we identified 91 peak regions as the DSDB set and 1035 peak regions as the DSNDB set. We collected 300 sequences that did not show binding by randomly chosen from the genome as negative data. In our DSDB experiment, we used 61 and 30 positive data along with 200 and 100 negative data as the training and testing data, respectively. In the DSNDB experiment we used 240 and 120 positive data with 200 and 100 negative data as the training and testing data, respectively. The four-fold cross validation method was performed to avoid the overfitting problem.

###### Epigenomic data preprocessing.

For each epi- data, we generated the density files from the mapped read files. The preprocessing procedure is to extend each aligned read to an assumed length of 200bp and then to count the number of extended fragments locating in every 25 nucleotide window in the genome [[8](#_ENREF_8)]. The counts were normalized to number of fragments per 10 million aligned reads. Then the intensity of a binding site was calculated as the average normalized counts across the site. Note that since methylation data have biases towards to CpG density, we preprocessed their read counts normalized against CpG density.

###### Recognition of TF binding sites.

We first scanned the sequence with the PSWM of the TFs under a very-low-threshold constraint to identify putative binding sites. Note that in theory every position should be considered a possible binding site (i.e. able to be bound by any TF). Here we adopted a very-low-threshold constraint, its PSWM score (the log-likelihood score) is bigger than the consensus motif PSWM score - 6, to filter out those very weak sites to make the computational tasks accomplishable by a 16-core-96G-RAM computer, while still allowing both strong and weak binding sites in the sequence.

###### Strong and weak Oct4 binding sites.

A binding site was called a strong Oct4 TFBS when its PSWM score (the log-likelihood score) was bigger than the consensus PSWM score – 2.5. A binding site was called a weak site when its PSWM matching score was between the consensus PSWM score – 2.5 and the consensus PSWM score - 6. Other thresholds were used to check the robustness of our finding, where strong and weak sites were identified as consensus PSWM score – 3.5 and consensus PSWM score – 7.0.

###### **References to supplementary methods**

1. Kasowski M, Grubert F, Heffelfinger C, Hariharan M, Asabere A, et al. (2010) Variation in transcription factor binding among humans. Science 328: 232-235.

2. McDaniell R, Lee BK, Song L, Liu Z, Boyle AP, et al. (2010) Heritable individual-specific and allele-specific chromatin signatures in humans. Science 328: 235-239.

3. Chen X, Xu H, Yuan P, Fang F, Huss M, et al. (2008) Integration of external signaling pathways with the core transcriptional network in embryonic stem cells. Cell 133: 1106-1117.

4. Kent WJ, Sugnet CW, Furey TS, Roskin KM, Pringle TH, et al. (2002) The human genome browser at UCSC. Genome Res 12: 996-1006.

5. Langmead B, Trapnell C, Pop M, Salzberg SL (2009) Ultrafast and memory-efficient alignment of short DNA sequences to the human genome. Genome Biol 10: R25.

6. Lieberman-Aiden E, van Berkum NL, Williams L, Imakaev M, Ragoczy T, et al. (2009) Comprehensive mapping of long-range interactions reveals folding principles of the human genome. Science 326: 289-293.

7. Ernst J, Kheradpour P, Mikkelsen TS, Shoresh N, Ward LD, et al. (2011) Mapping and analysis of chromatin state dynamics in nine human cell types. Nature 473: 43-49.

8. Mikkelsen TS, Xu Z, Zhang X, Wang L, Gimble JM, et al. (2010) Comparative epigenomic analysis of murine and human adipogenesis. Cell 143: 156-169.
